# Exploration of restless legs syndrome under the new concept: A review

**DOI:** 10.1097/MD.0000000000032324

**Published:** 2022-12-16

**Authors:** Zhao Liu, Ruiqian Guan, Limin Pan

**Affiliations:** a Heilongjiang University of Traditional Chinese Medicine, Harbin, Heilongjiang Province; b Second Affiliated Hospital of Heilongjiang University of Traditional Chinese Medicine, Harbin, Heilongjiang Province; c First Affiliated Hospital of Heilongjiang University of Traditional Chinese Medicine, Harbin, Heilongjiang Province.

**Keywords:** dopamine dysfunction, dopamine receptor agonists, gabapentin, gene locus, hypersensitive cortical end points, iron deficiency, iron therapy, opioids, Restless legs syndrome

## Abstract

Restless leg syndrome (Restless legs syndrome, RLS) is a common neurological disorder. The pathogenesis of RLS remains unknown, and recent pathophysiological developments have shown the contribution of various genetic markers, neurotransmitter dysfunction, and iron deficiency to the disease, as well as other unidentified contributing mechanisms, particularly chronic renal dysfunction. RLS enhancement syndrome is frequently observed in patients with RLS who have received long-term dopamine agonist therapy, manifesting as a worsening of RLS symptoms, usually associated with an increase in the dose of dopamine agonist. Some patients with RLS can adequately control their symptoms with non-pharmacological measures such as massage and warm baths. First-line treatment options include iron supplementation for those with evidence of reduced iron stores, or gabapentin or pregabalin, as well as dopamine agonists, such as pramipexole. Second-line therapies include opioids such as tramadol. RLS seriously affects the quality of life of patients, and because its pathogenesis is unclear, more biological evidence and treatment methods need to be explored.

## 1. Introduction

Restless legs syndrome (Restless legs syndrome, RLS)/Willis-Ekbom disease (WED) are common neurological disorders encountered in neurology. It is a disease that can affect both sleep and physical health and well-being, and is known to cause insomnia. Although it has a serious impact on human health and sleep, there is insufficient awareness of this disease among non-neurology professionals.^[1]^

In recent years, due to some pathophysiological breakthroughs, the attention paid to RLS/WED has increased; therefore, this article reviews the latest progress in the pathophysiology, diagnosis, and treatment of RLS/WED.

RLS is also known as WED because the disease was first described by Sir Thomas Willis in 1672, who pointed out that those suffering from RLS were “followed by a great disturbance and disturbance of the limbs, so that they could not sleep, as if they were in a most painful state.”^[1]^ The clinical definition of RLS has evolved further over the years, but its basic features remain consistent with those described by Sir Willis, namely irresistible restlessness and the urge to move one’s legs, often accompanied by unpleasant sensations. This syndrome was further named and defined by Dr Karl Ekbom in the mid-20th century,^[1]^ and in 1995, the International Restless Legs Syndrome Study Group (International Restless Leg Syndrome Research Group, IRLSSG) was established. The diagnostic features of RLS have been further explained and clarified^[2]^ and have recently been updated.^[2]^ Although other groups, such as the American Psychological Association, have established similar standards,^[3]^ the definition of the IRLSSG group is still the most widely accepted and is therefore the main standard covered by this review. In addition, in order to maintain consistency and avoid confusion, despite previous efforts to propose a new, less stigmatizing medical term for RLS to be replaced by a new term for WED disease, this label is less known and has failed to gain widespread use and acceptance in recent years. We mainly use RLS as the more well-known medical term in the remainder of this paper.^[1]^

## 2. Epidemiology

RLS is becoming increasingly common, with the highest quality epidemiological reports showing a common morbidity of between 5% and 10% in adults in Europe and North America, and a morbidity of clinically significant RLS of 2.7%,^[4]^ with the lowest prevalence in nonwhite populations, 1.4% in Shanghai,^[5]^ and 1.8% in Japan.^[5]^ However, only a few studies have assessed the morbidity of RLS, which has been reported in 0.8% to 2.2% of new cases per year in the general population.^[5]^ Women are more likely to be affected than men, and the risk of RLS increases as pregnancy progresses.^[6]^ The morbidity of RLS symptoms is also increasing in adults older than 40 years, with some studies estimating morbidity as high as 18% to 23% in the elderly.^[7]^ Interestingly, studies have also shown that RLS is common in children and adolescents, affecting 1% to 4% of this population.^[7]^

Although the morbidity of RLS is high, most patients with RLS have mild to moderate symptoms, while only 1% to 3% of patients have severe and frequent symptoms.

## 3. Clinical presentation and diagnosis

RLS1 has the following characteristics: irresistible leg impulses at rest, which can be partially or completely relieved by exercise, and symptoms that mainly occur at night. Although RLS primarily affects the legs, patients have described it in other body regions, such as the mouth, neck, arms, face, abdomen, and genitals.^[8,9]^ The diagnostic criteria for RLS, which were last updated by the IRLSSG in 2014, consist of 5 key characteristics that must be met to diagnose RLS, as follows:

Diagnostic criteria for RLS defined by IRLSSG^[5]^

(1) An irresistible urge to the legs is usually but not always accompanied by unpleasant and uncomfortable sensations in the legs.
(2) Symptoms begin or worsen during periods of rest or inactivity such as lying down or sitting.
(3) Partial or total relief of symptoms due to movement such as walking or stretching, at least if the activity continues.
(4) Symptoms occur only at night or at night or are more severe than during the day.
(5) The occurrence of these features is not entirely a primary symptom of other medical or behavioral conditions (e.g., muscle pain, venous congestion, leg edema, and arthritis).

In addition to these 5 basic clinical criteria, 4 supportive characteristics need to be used, especially in suspicious cases, which were also redefined by the IRLSSG in 2014, as follows:

(1) A large number of periodic leg movements (Periodic Leg Movement in Sleep, PLMS) can occur while asleep or awake, and there is no evidence of disease or drugs that may cause or increase these movements.

PLMS is common in RLS and is reported to occur in 80% to 89% of patients with RLS.^[5]^ They occur in other conditions, such as respiratory sleep apnea and rapid eye movement sleep behavior disorder, and are a side effect of many medications that are common in healthy older people. In 2016, a joint working group commissioned by the IRLSSG and the European Restless Legs Syndrome Research Group (European Restless Legs Syndrome Study Group) developed and revised the scoring criteria. The cutoff value (13/H–15/H) for PLMs associated with RLS was slightly modified.^[8]^

(1) Response to dopaminergic therapy. Approximately 60% to 75% of patients^[6]^ have a good initial response to dopaminergic drugs. In general clinical practice, the lack of response to dopaminergic therapy is of concern for diagnostic accuracy, but the diagnosis of RLS is not necessarily excluded.^[9]^
(2) Family history. Having a first-degree relative with RLS increases the risk of RLS by 6 to 7 times.^[10]^
(3) There is a lack of an expected level of sleepiness during the day.^[10]^ This is especially true in moderate-to-severe cases.

It should be noted that PLMS during sleep is considered a supportive criterion for the diagnosis of RLS, but it is neither a necessary nor sufficient condition for the diagnosis of RLS. Nevertheless, PLMS are present in more than 80% of patients with RLS. PLMS is also common in patients without symptoms of RLS and is found in approximately 7.63% to 25% of the general population.^[11]^ A common misconception about RLS is that it is synonymous with periodic limb movement disorder (Periodic Limb Movement Disorder, PLMD), so PLMD or occasionally isolated PLMS is often misdiagnosed as RLS. The diagnosis of PLMD is limited to patients without RLS, in whom periodic limb movements during sleep are the primary sleep disorders associated with symptoms of insomnia or sleep deprivation. The diagnosis of PLMD is strictly dependent on polysomnography and a history of related sleep disturbances, whereas the diagnosis of RLS does not require a polysomnography.^[12]^

## 4. Pathophysiology

Most reports of families with RLS are consistent with the typical autosomal dominant inheritance pattern, with different manifestations.^[13]^ In addition, data from a large family study showed vertical transmission in 90% of 671 RLS families studied, indicating a dominant mode of inheritance.^[14]^ However, lineages with recessive inheritance patterns have also been reported. Interestingly, according to a family report, 2.8% of families show bilinear inheritance.^[14]^

Phenotypic duplication, that is, individuals with similar manifestations due to different environmental, genetic, or both determinants, may occur in RLS patients and RLS families, which is relatively frequent in RLS patients and RLS families,^[15]^ but the occurrence of this situation can be explained by chance. Some reports appear to follow a non-Mendelian pattern of inheritance because, despite the apparent autosomal dominant mode of inheritance, the proportion of those affected (>50%) is higher than would be expected from typical autosomal dominant inheritance. Zimprich^[3]^ proposed the possible role of genetic factors, trying to explain the characteristics of non-Mendelian inheritance patterns in RLS genetics and the existence of epigenetic inheritance in this disease family.

Many studies have attempted to identify the genes involved in the basic molecular mechanisms of this disease, but no single cause of RLS has been identified.^[3]^ To date, genome-wide association studies of RLS have identified 13 different genes in several communities in Northern Europe, These genes are represented by chromosome 6p21.2 (BTBD9), 2p14 (MEIS1), 9p24.1-p23 (PTPRD), 15q23 (MAP2K5/SKOR1), and 16q12.1 (TOX3/BC034767) mononucleotides.^[16,17]^ In addition, recent studies have identified 7 additional major susceptibility loci for RLS: RLS1 at 12q12-q21, RLS2 at 14q13-q21, RLS3 at 9p24-p22, RLS4 at 2q33, RLS5 at 20p13, RLS6 at 19p13, and RLS7 at 16p12.1.^[18,19]^ The pathophysiological functions of these genomic loci are yet to be defined, but their main functions appear to be related to the neural development of the limb during the embryonic period.^[11]^ However, variation at these genomic loci accounts for only a small fraction of genetic susceptibility to RLS.

### 4.1. The iron deficiency hypothesis

Ekbom and Nordlander have both investigated the role of iron in the early stages of RLS disease,^[20,21]^ and several imaging studies have identified a close relationship between iron metabolism and RLS, particularly in the brain.^[22]^ It is not clear whether RLS is related to low iron levels in the peripheral or central nervous system (CNS). Early evidence suggests that the severity of RLS increases with a decrease in peripheral iron, which may be related to the level of peripheral iron^[23]^ and that the morbidity of RLS is higher in patients with peripheral iron deficiency.^[24]^ However, recent findings have challenged these findings, with a population-based study establishing that RLS is not associated with plasma ferritin levels.^[25]^ Another study suggested that the morbidity of RLS in iron-deficient patients has not been demonstrated in population-based cross-sectional studies.^[26]^ Currently, the prevailing view is that iron deficiency in the brain is a key biological driver of RLS and may be due to various factors including low peripheral iron levels or genetic factors.^[27]^ Allen stated in 2015 that “the pathophysiology of RLS appears to involve a regional brain defect present in the majority of patients with RLS despite a normal iron status.”^[6]^

One proposed pathway for CNS iron content to mediate the pathophysiology of RLS is through pathways that activate a hypoxic state. Elevated levels of hypoxia-inducible factor 1-α in substantia nigra neurons, as well as elevation of hypoxia-inducible factor 2-α and vascular endothelial growth factor, were observed in microvessels of RLS patients.^[28]^ Hypoxia in peripheral tissues is also believed to be the cause of RLS symptoms, and iron deficiency may lead to the activation of the hypoxia pathway, which may affect the regulatory transport mechanism of iron in the blood-brain barrier^[29]^ because oxygen transport requires iron to be effective.^[29]^ However, the pathway of the peripheral hypoxic state has also been implicated in the pathophysiology of RLS and the generation of symptoms. One study showed that skin measurements of peripheral tissue hypoxemia were strongly correlated with the severity of symptoms of RLS, and that both symptoms and hypoxemia were partially reversed by dopaminergic therapy.^[29]^ The exact causal relationship between the reduction of the CNS and hypoxic state is unclear, and a 2-way relationship is considered most likely.^[30]^

### 4.2. Targeting hypersensitive cortical end points in RLS

Brain iron deficiency (BID) has been recognized as a major initial pathogenesis in the development of RLS. This is based on extensive research using cerebrospinal fluid (CSF), autopsy materials, and brain imaging. This suggests a regional reduction in brain iron^[31,32]^ and is further supported by the efficacy of iron supplementation for RLS,^[33]^ including otherwise refractory RLS. Animal models have established a causal relationship between BID and altered dopamine function in RLS. Changes in the dopamine system produced during BID after weaning in rats and mice are similar to those found in RLS and thus represent a valuable pathophysiological model for RLS.^[34]^

Recent studies have shown that brain iron deficiency in rodents is associated with specific changes in adenosine neurotransmitters, which may provide a pathological link between PLMS in BID and RLS, and glutamate mechanisms involved in excessive anxiety.^[35]^ These include changes in the density of striatal adenosine receptors that regulate corticostriatal glutamate release, which are expected to increase corticostriatal glutamate neurotransmitter sensitivity. Therefore, it can be speculated that increased sensitivity of striatal glutamate neurotransmitters may be a pathogenic cause of PLMS development in RLS.^[36]^

Intercortical glutamate terminal hypersensitivity was demonstrated in rodents with BID using a recently introduced in vivo optogenetic-microdialysis approach.^[37]^ Notably, pramipexole, ropinirole, and gabapentin target highly sensitive and control glutamate terminals. The involvement of the D2 and D4 receptors in the action of pramipexole has been demonstrated using specific dopamine receptor antagonists. In particular, it has been suggested that D4 receptor selective agonists may provide better treatment for RLS.^[38]^

### 4.3. Neurotransmitter dysfunction

The almost immediate improvement of RLS symptoms with small doses of most dopaminergic agents supports the pathophysiological role of the dopamine system; however, the opposite effect, exacerbation of symptoms, occurs with dopamine antagonists.^[38]^

The pathophysiological mechanisms leading to these results are unknown, and dopaminergic agents may improve RLS symptoms because of their effects on neural networks rather than correcting dopamine deficiency.^[39]^ Dopaminergic agonists need to cross the blood-brain barrier to relieve RLS symptoms, suggesting that dopamine plays a role in the CNS rather than in the peripheral nervous system in the pathophysiology of RLS.^[40]^ However, most preclinical and clinical evidence suggests that the dopaminergic system is highly active, with highly sensitive corticocerebral pathways. RLS includes both motor and sensory symptoms. Both sensory and motor circuits and their interactions in sensorimotor integration are under descending control of the monoaminergic population in the brain. These clusters with spinal projections are the dorsal area (serotonin), thalamic area (norepinephrine), and A11 area behind the hypothalamus (dopamine).^[16]^ Some researchers have hypothesized that dopaminergic A11 cells play an important role in RLS pathophysiology. In mice, bilateral damage to the A11 nucleus results in hyperactivity.^[41]^

However, this hypothetical basis for RLS has not been shown to exist directly in humans, as anatomical studies of patients with RLS have shown no abnormalities in the A11 dopaminergic system.^[42]^

Although treatment with an evidential dopaminergic agonist may initially alleviate RLS symptoms, given the frequency of potentiating syndromes, these agents may not be a long-term solution. Enhancement syndrome is often seen in patients with RLS who are receiving long-term dopamine agonist therapy. It is characterized by a worsening of RLS symptoms, usually associated with an increase in the dose of dopamine agonists.^[43]^ Enhancement syndrome has the following criteria.

(1) A temporal relationship was observed between the increase in the overall intensity of the moving impulse or sensation and the increase in the daily dose of medication.
(2) There was a temporal relationship between the decrease in the overall intensity of the impulse to move or sensation and the decrease in the daily dose of medication.
(3) The latency to develop symptoms of RLS at rest was shorter than the latency to respond to the initial treatment or before starting treatment.
(4) Impulse or sensation of movement extends to a previously unaffected limb or body part.
(5) The duration of treatment effect was shorter than that of the initial treatment response.
(6) Periodic limb movements in wakefulness for the first time or more severe than in response to initial treatment or prior to initiation of treatment.

Alterations in the dopaminergic system, that is, the presynaptic hyperdopaminergic state, appear to be involved in causing PLMS, and alterations in the glutamate neurotransmitter, that is, the presynaptic hyperglutamate state, are involved in causing excessive anxiety and PLMS.^[41,44,45]^

Furthermore, it has been shown in animal models that iron deficiency in the brain leads to downregulation of adenosine A1 receptors in the striatum and cortex and, secondarily, to a decrease in adenosinergic extracellular content.^[46,47]^ Ultimately, decreased adenosinergic activation may lead to increased intercortical dopaminergic and glutamatergic activity in RLS^[6,46]^ This hypoadenylic state provides a mechanism to explain PLMS, that is, hypersensitivity secondary to cortical terminals.^[47]^ However, it may also explain excessive anxiety secondary to A1 receptor downregulation in the cortex, basal forebrain, or hypothalamus.^[48]^ The therapeutic efficacy of dipyridamole, a drug that blocks the balanced nucleoside transporter 1 (Equilibrium Nucleoside Transporter 1, ENT-1) reuptake mechanism in humans, thereby increasing extracellular adenosine, lends plausibility to this hypothesis.^[49]^

## 5. Therapeutics

### 5.1. Overall management principles and methods

For patients with intermittent or mild symptoms of RLS, non-pharmacological treatment is required, which may be the only treatment necessary. However, for patients with other types of RLS, non-pharmacological measures should also be considered and recommended as complementary treatment to prescription medication. Non-pharmacological treatments that can be helpful for RLS include massage, stretching, walking, cognitive distraction, or warm or cold baths.^[50]^ Although these measures are generally tolerable and safe for all patients with RLS, the benefits of non-drug treatments are often short-lived and lack conclusive evidence. There are low-level evidence trials on measures such as exercise,^[51]^ yoga,^[52]^ and lavender oil massage.^[53]^ These non-pharmacological treatments may also be recommended as adjunctive therapies, possibly helping to avoid higher doses of medication.

In addition, the general principle of the initial RLS treatment approach, especially for those with intermittent or mild disease, is to select an appropriate monotherapy strategy to treat RLS, which may help avoid adverse effects and reduce the possibility of drug-drug interactions.

Patients with chronic refractory RLS symptoms should take prescription medication daily, and the choice of medication should consider that comorbidities and symptoms precede the patient’s habitual RLS symptoms. In general, a single daily dose is sufficient, but some patients need to take the medicine in divided doses in advance to fully cover the disturbing symptoms during the day. This is more common in the later stages of chronic refractory RLS, especially in patients whose symptoms evolve earlier.

Patients with sufficiently mild symptoms can often be effectively managed with alpha-2-delta ligand drugs (e.g., gabapentin and pregabalin), while those with moderate or severe symptoms may require dopaminergic therapy or consideration of opioids. In the case of refractory RLS, sequential rotation of monotherapy using different drug classes should be attempted. In the case of augmentation, dose reduction or complete reduction of dopaminergic drugs is helpful.^[7]^

Treatment with alternative drug classes, such as alpha-2-delta ligand drugs or opioids, for those patients who appear to have developed resistance to a drug, one way to consider this is a “drug holiday,”^[54]^ that is, to try to reduce the current drug therapy because the apparent resistance to this drug has developed and the relief effect on RLS symptoms has diminished. It may be substituted with or without another class of drugs, and a previously effective drug may be restored or reintroduced. However, to the best of our knowledge, there is no evidence to guide this practice, and further detailed management methods and algorithms are found in the RLS/WED Foundation consensus statement on RLS management.^[1]^

### 5.2. Iron therapy

The iron deficiency hypothesis remains the most important in the pathophysiology of RLS, although oral and intravenous iron therapies have different efficacies in the treatment of RLS. Peripheral iron storage should be evaluated at the time of the initial diagnosis of RLS and later during long-term treatment whenever there is a change in symptom control, particularly if the frequency or severity of symptoms is characterized by an increase or an overall clinical deterioration, or if the response to previously effective therapy is diminished. If iron stores are low, iron replacement therapy should generally be used either as monotherapy (if symptoms are relatively mild) or in combination with another RLS treatment.

If iron replacement therapy is required in patients with RLS, the first-line approach is oral iron replacement therapy. Currently, the most common oral iron supplementation therapy is ferrous sulfate. Supplementary vitamin C contributes to the absorption of iron in the digestive tract and often minimizes adverse reactions. Alternatively, a ferrous fumarate preparation may be considered, with a dose of 65 mg of elemental iron administered approximately 1 h before a meal to promote optimal absorption.

Intravenous iron supplementation may be considered if oral iron is not tolerated or effectively absorbed, and in patients with severe RLS symptoms, there are currently several formulations with the best evidence supporting the use of carboxymethyl iron, with a total dose of 1000-1500 mg in single or fractional infusions.^[55]^

### 5.3. The gabapentin/α-2-δ ligand drug

Gabapentin^[56]^ and related drugs have recently become the first-line agents of choice for the treatment of RLS. The mechanism of action of alpha-2-delta drugs is to affect the release of neurotransmitters by binding to alpha-2-delta calcium channel receptors in presynaptic neurons. Gabapentin is named after its structural similarity to the gamma-aminobutyric acid (GABA) neurotransmitter, although there is no direct effect of gabapentin or related compounds on GABA receptor regulation. Gabapentin was later used to treat RLS, although it was not approved by the Food and Drug Administration for this disease. Moreover, relatively few trials have specifically tested gabapentin for RLS, although small randomized trials have demonstrated its efficacy.^[57]^

Gabapentin can be started at a dose of 100 to 300 mg and taken 1 to 2 hours before the onset of evening symptoms. And may be administered in divided doses throughout the day depending on whether the patient has evening or morning symptoms (i.e., twice daily, 3 times daily, or 4 times daily depending on the patient’s symptoms and perceived time of action) and then incrementally at a rate of 100 to 300 mg as needed and tolerated, usually once every 3 to 7 days, to a broad target dose of 600 to 2400 mg per night, This was rigorously analyzed in an earlier proof-of-concept, randomized, double-blind, and placebo-controlled crossover trial, which showed a mean effective dose of gabapentin of 1855 mg/day and an effective dose of 1391 to 2400 mg/day.^[58]^

Lyrica^[59]^ may be of particular consideration in patients who respond to and tolerate gabapentin but require a higher dose to achieve efficacy, as there is no dose limit for its absorption, and its mechanism of action is similar to that of gabapentin. There is ample evidence that pregabalin has therapeutic effects on RLS. A pivotal RCT compared the efficacy of pregabalin and pramipexole and found that a daily dose of 300 mg pregabalin provided comparable or better efficacy than pramipexole.^[60]^

### 5.4. Dopaminergic therapy

Dopamine agonists (pramipexole, ropinirole, and rotigotine) are the traditional mainstay of treatment for RLS. Initially, carbidopa/levodopa was found to be effective in relieving restless leg symptoms, which inspired the dopamine hypothesis for RLS. Subsequently, through a series of randomized controlled trials of specialized clinical development programs, the food and drug administration made dopamine agonists suitable drugs for the treatment of RLS.^[61]^

However, over the past decade, there has been a growing body of evidence suggesting that dopaminergic therapy has several drawbacks owing to its potential for adverse events. Most importantly, enhancement syndrome occurs in a significant proportion of RLS patients treated with excessive dopaminergic therapy, with a morbidity rate of approximately 8% per year.^[50]^ As previously mentioned, potentiation syndrome represents the progression of RLS symptoms in time and space, with symptoms occurring earlier and tending to last a day; symptoms of increasing intensity occur at shorter latencies, become highly sleep disordered, and involve varying degrees of spread of symptoms from the legs to other areas of the body, particularly the arms. Another important concern with dopamine drugs is the frequency of impulse control disorder symptoms, which occur in approximately 15% of patients (compared to 6%–8% in patients with sleep disorders not treated with dopamine agonists), including a range of undesirable behaviors, such as compulsive and financially disruptive shopping, gambling, mischief, and other quasi-addictive behaviors.^[62]^ A common theme in impulse control disorder spectrum behaviors is the inability to control impulses toward undesirable behaviors, often leading to socially destructive consequences.

In addition, adverse dose-related effects, such as dizziness, drowsiness, nausea, or headache, may occur with dopamine agonists and can usually be relieved by reducing the dose or discontinuing medication. A rare but potentially serious adverse effect of dopamine agonists is excessive daytime drowsiness with sleep episodes, leading to harmful consequences, such as drowsy driving or motor vehicle crashes.^[63]^

Pramipexole (Mirapex) starts at 0.125 mg and is taken approximately 1 to 2 hours before bedtime. It can then be increased by 0.125 mg every 3 to 7 days to achieve the target dose in the range of 0.375 to 0.5 mg, with the lowest effective dose to control symptoms. The maximum dose of pramipexole is in the range of 0.5 mg/day for some patients and should only be exceeded in rare cases because of the increased risk of potentiation syndrome at higher doses.^[64]^

Another option is ropinirole (Requip), with an initial dose of 0.25 mg, in increments of 0.25 to 0.5 mg, and a maximum daily dose range of 3.0 to 4.0 mg. However, some experts have considered more aggressive use of the drug in the 4.0 to 6.0 mg per day dose range, with similar careful observation and adequate counseling of patients.

Rotigotine (Neupro) patches can provide patients with round-the-clock symptom control because of their continuous, progressive, all-day release.^[65]^ This extended duration of action and sustained release of dopamine into the blood may minimize dopamine fluctuations at the receptor and may be associated with a low propensity to evolve into potentiation syndrome.^[65]^ The dose of rotigotine can be started at a patch strength of 1.0 mg and increased weekly to a maximum daily dose of 2.0 or 3.0 mg.^[66]^ Clinical trials of rotigotine have shown that a further dose increase to 4.0 mg per day could be considered, but this high dose may carry a greater risk of enhancement.^[67]^ Rotigotine, as a transdermal patch, may cause skin reactions, such as excessive itching or redness of the skin at the application site. However, this could be avoided or minimized by rotating the patch position daily.

4.5 Opioid therapy Evidence-based opioid therapy is effective for the treatment of RLS, particularly long-acting oxycodone-naloxone. Opioids are often reserved for patients who have failed other medications.^[68]^ With careful supervision and appropriate counseling, chronic opioid therapy is appropriate for selected RLS.

In the treatment of chronic severe RLS symptoms, an initial dose of opioids followed by an escalation to a higher dose is the preferred approach, unless the patient has particularly severe symptoms or late enhancement syndrome, which may require an escalation to a higher dose of opiates. Tramadol, 50 to 200 mg per night must not exceed and be further escalated to a maximum daily dose of 300 to 400 mg, usually an initial low-dose opioid for the treatment of RLS.

The next intermediate-acting opioid to treat RLS is oxycodone, starting at 5 to 10 mg and increasing in doses of 5 to 10 mg every night, 1 h before the onset of symptoms. In addition, patients with early daytime symptoms can take it 2 to 3 times a day according to a schedule of once every 6 to 12 hours.

## 6. Discussion and future outlook

The past 5 years have seen a remarkable convergence of clinical and laboratory research on RLS. Clinical studies have shown the need for refinement of the diagnostic criteria and definition of motor signs of RLS and have provided relevant data, namely PLMS. The observation of iron deficiency in the brains of patients with RLS provides a basis for exploring several areas of research. Studies of the effects of iron deficiency on the spinal dopamine system and brain have suggested that adenosine may play an important role in the disease. Clinical and animal studies have demonstrated a putative link between RLS risk alleles and iron^[69]^ and basal ganglia development.^[29]^ Despite the remarkable convergence phenomena,

The biggest challenge is finding new treatments, as most patients report inadequate long-term treatments. More immediately, commonly used combination therapies should be evaluated. Clinical trials of combination therapy are needed that take into account knowledge of the pathophysiology of the spine and metabolic pathways, as well as known drug side effects.

Therefore, there is still a long way to go in researching RLS.

## Author contributions

**Conceptualization:** Zhao Liu.

**Data curation:** Zhao Liu, Ruiqian Guan, Limin Pan.

**Formal analysis:** Zhao Liu.

**Funding acquisition:** Zhao Liu.

**Investigation:** Zhao Liu.

**Methodology:** Zhao Liu.

**Project administration:** Zhao Liu.

**Resources:** Zhao Liu.

**Software:** Zhao Liu.

**Supervision:** Zhao Liu.

**Validation:** Zhao Liu.

**Visualization:** Zhao Liu.

**Writing – original draft:** Zhao Liu.

**Writing – review & editing:** Zhao Liu.

## References

[1] Gossard T Trotti L Videnovic A. Restless legs syndrome: contemporary diagnosis and treatment. Neurotherapeutics. 2021;18:140–55. 33880737 10.1007/s13311-021-01019-4 PMC8116476

[2] Mazurie Z Mayo W Ghorayeb I. Attention-deficit/hyperactivity and obsessive-compulsive symptoms in adult patients with primary restless legs syndrome. Appl Neuropsychol Adult. 2022;1:8. 10.1080/23279095.2022.2057857 35382650

[3] Jiang Y Fann C Fuh J. Genome-wide analysis identified novel susceptible genes of restless legs syndrome in migraineurs. J Headache Pain. 2022;23:39. 35350973 10.1186/s10194-022-01409-9 PMC8966278

[4] Trenkwalder C Tinelli M Sakkas G. Socioeconomic impact of restless legs syndrome and inadequate restless legs syndrome management across European settings. Eur J Neurol. 2021;28:691–706. 33043569 10.1111/ene.14582

[5] Gonzalez-Latapi P Malkani R. Update on restless legs syndrome: from mechanisms to treatment. Curr Neurol Neurosci Rep. 2019;19:54. 31250128 10.1007/s11910-019-0965-4

[6] Ferré S García-Borreguero D Allen R. New insights into the neurobiology of restless legs syndrome. Neuroscientist. 2019;25:113–25. 30047288 10.1177/1073858418791763 PMC9372713

[7] Weiss H. Safety of dopamine agonists for treating restless legs syndrome. Mov Disord. 2019;34:150. 10.1002/mds.27573 30653731

[8] Silber M Buchfuhrer M Earley C. The management of restless legs syndrome: an updated algorithm. Mayo Clin Proc. 2021;96:1921–37. 34218864 10.1016/j.mayocp.2020.12.026

[9] Lai Y Hsieh K Cheng Y. Striatal histamine mechanism in the pathogenesis of restless legs syndrome. Sleep. 2020;43:zsz223. 31671173 10.1093/sleep/zsz223 PMC8491621

[10] Marshall N Serinel Y Killick R. Magnesium supplementation for the treatment of restless legs syndrome and periodic limb movement disorder: a systematic review. Sleep Med Rev. 2019;48:101218. 31678660 10.1016/j.smrv.2019.101218

[11] Akçimen F Ross J Sarayloo F. Genetic and epidemiological characterization of restless legs syndrome in Québec. Sleep. 2020;43:sz265. 10.1093/sleep/zsz265 31665514

[12] Dafkin C Mckinon W Kerr S. Restless legs syndrome: clinical changes in nervous system excitability at the spinal cord level. Sleep Med Rev. 2019;47:9–17. 31212170 10.1016/j.smrv.2019.05.005

[13] Wang B Duanyang L Jie W. Meta-analysis of single nucleotide polymorphisms in restless legs syndrome risk genes. Chin J Behav Med Brain Sci. 2022;31:187–92.

[14] Wang B Wei W. Research progress on single nucleotide polymorphisms of restless legs syndrome risk genes. Chin J Neurol. 2021;54:1187–93.

[15] Zhao X Ren J Sun S. Clinical features and standardized treatment of restless legs syndrome. Chin J Clin Pharmacol Ther. 2021;26:497–503.

[16] Kocar T Müller H Kassubek J. Differential functional connectivity in thalamic and dopaminergic pathways in restless legs syndrome: a meta-analysis. Ther Adv Neurol Disord. 2020;13:1756286420941670. 32821291 10.1177/1756286420941670 PMC7412904

[17] Akçimen F Sarayloo F Liao C. Transcriptome-wide association study for restless legs syndrome identifies new susceptibility genes. Commun Biol. 2020;3:373. 32651461 10.1038/s42003-020-1105-z PMC7351781

[18] Lin W. The conundrum of the origin of restless legs syndrome. Sleep. 2022;45:zsac018. 35554576 10.1093/sleep/zsac018

[19] Estiar M Senkevich K Yu E. Lack of causal effects or genetic correlation between restless legs syndrome and Parkinson’s disease. Mov Disord. 2021;36:1967–72. 33974305 10.1002/mds.28640 PMC9017317

[20] Lv Q Wang X Asakawa T. Pharmacologic treatment of restless legs syndrome. Curr Neuropharmacol. 2021;19:372–82. 33380302 10.2174/1570159X19666201230150127 PMC8033969

[21] Badenoch J Searle T Watson I. Sensory symptoms in body-focused repetitive behaviors, restless legs syndrome, and Tourette syndrome: an overlap? Neurosci Biobehav Rev. 2020;119:320–32. 33086129 10.1016/j.neubiorev.2020.10.008

[22] Tuovinen N Stefani A Mitterling T. Functional connectivity and topology in patients with restless legs syndrome: a case-control resting-state functional magnetic resonance imaging study. Eur J Neurol. 2021;28:448–58. 33032390 10.1111/ene.14577 PMC7820983

[23] Lammers N Curry-Hyde A Smith A. Are serum ferritin and transferrin saturation risk markers for restless legs syndrome in young adults? Longitudinal and cross-sectional data from the Western Australian Pregnancy Cohort (Raine) Study. J Sleep Res. 2019;28:e12741. 30062860 10.1111/jsr.12741

[24] Guidelines for the diagnosis and treatment of restless legs syndrome in China (2021 edition). Chin Med J (Engl). 2021;101:908–25.

[25] Olgun Yazar H Yazar T Özdemir S. Serum C-reactive protein/albumin ratio and restless legs syndrome. Sleep Med. 2019;58:61–5. 31129525 10.1016/j.sleep.2019.02.022

[26] Pennestri M Petit D Paquet J. Childhood restless legs syndrome: a longitudinal study of prevalence and familial aggregation. J Sleep Res. 2021;30:e13161. 32783271 10.1111/jsr.13161

[27] Türkoglu S Bolac E Yildiz S. Presynaptic inhibition in restless legs syndrome. Int J Neurosci. 2021;131:213–9. 32108535 10.1080/00207454.2020.1737048

[28] Romero-Peralta S Cano-Pumarega I García-Borreguero D. Emerging concepts of the pathophysiology and adverse outcomes of restless legs syndrome. Chest. 2020;158:1218–29. 32247713 10.1016/j.chest.2020.03.035

[29] Haschka D Volani C Stefani A. Association of mitochondrial iron deficiency and dysfunction with idiopathic restless legs syndrome. Mov Disord. 2019;34:114–23. 30311259 10.1002/mds.27482

[30] Ferri R Delrosso L Silvani A. Peculiar lifespan changes of periodic leg movements during sleep in restless legs syndrome. J Sleep Res. 2020;29:e12896. 31313413 10.1111/jsr.12896

[31] Garcia-Malo C Novo-Ponte S Castro-Villacañas Farzamnia A. Correlation between systemic iron parameters and substantia nigra iron stores in restless legs syndrome. Sleep Med. 2021;85:191–5. 34343769 10.1016/j.sleep.2021.07.027

[32] Caixia Y Liuqing W Shoucheng Z. Progress in diagnosis and treatment of restless legs syndrome. Chin J Gen Pract. 2019:994–7.

[33] Lyu S Doroodchi A Xing H. BTBD9 and dopaminergic dysfunction in the pathogenesis of restless legs syndrome. Brain Struct Funct. 2020;225:1743–60. 32468214 10.1007/s00429-020-02090-x PMC7429108

[34] Salminen A Silvani A Allen R. Consensus guidelines on rodent models of restless legs syndrome. Mov Disord. 2021;36:558–69. 33382140 10.1002/mds.28401 PMC8313425

[35] Shin J Lee J Kim H. Bioinformatic analysis of proteomic data for iron, inflammation, and hypoxic pathways in restless legs syndrome. Sleep Med. 2020;75:448–55. 32992101 10.1016/j.sleep.2020.09.002

[36] Chawla S Gulyani S Allen R. Extracellular vesicles reveal abnormalities in neuronal iron metabolism in restless legs syndrome. Sleep. 2019;42:zsz079. 30895312 10.1093/sleep/zsz079 PMC6612672

[37] Zhang R Werner A Hermann W. Thalamic GABA may modulate cognitive control in restless legs syndrome. Neurosci Lett. 2019;712:134494. 31520647 10.1016/j.neulet.2019.134494

[38] Lopez R Micoulaud Franchi J Chenini S. Restless legs syndrome and iron deficiency in adults with attention-deficit/hyperactivity disorder. Sleep. 2019;42:zsz027. 30722056 10.1093/sleep/zsz027

[39] Kim J Hartzema A. Decline in dopamine agonists prescribing and characteristics of drugs prescribed during ambulatory office visits for restless legs syndrome: the national ambulatory medical care survey 2007-2015. Sleep Med. 2019;54:238–43. 30590306 10.1016/j.sleep.2018.11.011

[40] Avni T Reich S Lev N. Iron supplementation for restless legs syndrome—a systematic review and meta-analysis. Eur J Intern Med. 2019;63:34–41. 30798983 10.1016/j.ejim.2019.02.009

[41] Dulski J Wąż P Konkel A. The impact of subthalamic deep brain stimulation on restless legs syndrome in Parkinson’s disease. Neuromodulation. 2021. 10.1111/ner.13462 34036673

[42] Earley CJ Allen RP Connor JR. The dopaminergic neurons of the A11 system in RLS autopsy brains appear normal. Sleep Med. 2009;10:1155–7. 19307154 10.1016/j.sleep.2009.01.006 PMC2783651

[43] Earley CJ Connor J Garcia-Borreguero D. Altered brain iron homeostasis and dopaminergic function in Restless Legs Syndrome (Willis–Ekbom Disease). 10.1016/j.sleep.2014.05.009 25201131

[44] Klepitskaya O. Author response: deep brain stimulation improves restless legs syndrome in patients with Parkinson disease. Neurology. 2019;92:869. 31036575 10.1212/WNL.0000000000007412

[45] Alonso-Navarro H García-Martín E Agúndez J. Association between restless legs syndrome and other movement disorders. Neurology. 2019;92:948–64. 31004074 10.1212/WNL.0000000000007500

[46] Tilch E Schormair B Zhao C. Identification of restless legs syndrome genes by mutational load analysis. Ann Neurol. 2020;87:184–93. 31788832 10.1002/ana.25658

[47] Lyu S Deandrade M Unger E. Mu opioid receptor knockout mouse: phenotypes with implications on restless legs syndrome. J Neurosci Res. 2020;98:1532–48. 32424971 10.1002/jnr.24637 PMC7430552

[48] Sheng L Zhao P Ma H. Grey matter alterations in restless legs syndrome: a coordinate-based meta-analysis. J Sleep Res. 2021;30:e13298. 33554365 10.1111/jsr.13298

[49] Garcia-Borreguero D Garcia-Malo C Granizo J. A randomized, placebo-controlled crossover study with Dipyridamole for restless legs syndrome. Mov Disord. 2021;36:2387–92. 34137476 10.1002/mds.28668 PMC8530834

[50] Buchfuhrer M Baker F Singh H. Noninvasive neuromodulation reduces symptoms of restless legs syndrome. J Clin Sleep Med. 2021;17:1685–94. 33949942 10.5664/jcsm.9404 PMC8656897

[51] Song M Park K Motamedi G. Cognitive behavioral therapy for insomnia in restless legs syndrome patients. Sleep Med. 2020;74:227–34. 32861015 10.1016/j.sleep.2020.07.011

[52] Innes K Selfe T Montgomery C. Effects of a 12-week yoga versus a 12-week educational film intervention on symptoms of restless legs syndrome and related outcomes: an exploratory randomized controlled trial. J Clin Sleep Med. 2020;16:107–19. 31957638 10.5664/jcsm.8134 PMC7053002

[53] Bonakis A Androutsou A Koloutsou M. Restless legs syndrome masquerades as chronic insomnia. Sleep Med. 2020;75:106–11. 32858348 10.1016/j.sleep.2020.06.003

[54] Silber MH Becker PM Earley C. Willis-Ekbom disease foundation revised consensus statement on the management of restless legs syndrome. Mayo Clin Proc. 2013;88:977–86. 24001490 10.1016/j.mayocp.2013.06.016

[55] Garcia-Malo C Miranda C Novo Ponte S. Low risk of iron overload or anaphylaxis during treatment of restless legs syndrome with intravenous iron: a consecutive case series in a regular clinical setting. Sleep Med. 2020;74:48–55. 32841843 10.1016/j.sleep.2020.06.002

[56] Garcia-Borreguero D Kohnen R Silber MH. The longterm treatment of restless legs syndrome/Willis-Ekbom disease: evidence-based guidelines and clinical consensus best practice guidance: a report from the international restless legs syndrome study group. Sleep Med. 2013;14:675–84. 23859128 10.1016/j.sleep.2013.05.016

[57] Garcia-Borreguero D Cano-Pumarega I Garcia Malo C. Reduced response to gabapentin enacarbil in restless legs syndrome following long-term dopaminergic treatment. Sleep Med. 2019;55:74–80. 30772697 10.1016/j.sleep.2018.11.025

[58] Manconi M Garcia-Borreguero D Schormair B. Restless legs syndrome. Nat Rev Dis Primers. 2021;7:80. 34732752 10.1038/s41572-021-00311-z

[59] Garcia-Borreguero D Silber MH Winkelman JW. Guidelines for the frst-line treatment of restless legs syndrome/WillisEkbom disease, prevention and treatment of dopaminergic augmentation:a combined task force of the IRLSSG, EURLSSG, and the RLS-foundation. Sleep Med. 2016;21:1–11. 27448465 10.1016/j.sleep.2016.01.017

[60] Massey T Robertson N. Restless legs syndrome: causes and consequences. J Neurol. 2020;267:575–7. 31912211 10.1007/s00415-019-09682-6 PMC6989584

[61] Winkelman J. High national rates of high-dose dopamine agonist prescribing for restless legs syndrome. Sleep. 2022;45:zsab212. 34417810 10.1093/sleep/zsab212 PMC8842153

[62] Chenini S Barateau L Guiraud L. Depressive symptoms and suicidal thoughts in restless legs syndrome. Mov Disord. 2022;7:812–25. 10.1002/mds.28903 34985142

[63] Hankin C Lee D Garcia-Borreguero D. Increased risk for new-onset psychiatric adverse events in patients with newly diagnosed primary restless legs syndrome who initiate treatment with dopamine agonists: a large-scale retrospective claims matched-cohort analysis. J Clin Sleep Med. 2019;15:1225–32. 31538593 10.5664/jcsm.7908 PMC6760417

[64] Sakurai A Wakuda T Yoshida R. Successful discontinuation of oxycodone under pramipexole treatment for restless legs syndrome due to withdrawal. Psychiatry Clin Neurosci. 2021;75:112–3. 33289253 10.1111/pcn.13184

[65] Chenini S Rassu A Barateau L. Increased blood pressure dipping in restless legs syndrome with Rotigotine: a randomized trial. Mov Disord. 2020;35:2164–73. 32875658 10.1002/mds.28224

[66] Zhou X Du J Liang Y. The efficacy and safety of pharmacological treatments for restless legs syndrome: systemic review and network meta-analysis. Front Neurosci. 2021;15:751643. 34764852 10.3389/fnins.2021.751643 PMC8576256

[67] Muramatsu K Chikahisa S Shimizu N. Rotigotine suppresses sleep-related muscle activity augmented by injection of dialysis patients’ sera in a mouse model of restless legs syndrome. Sci Rep. 2019;9:16344. 31704978 10.1038/s41598-019-52735-z PMC6841937

[68] Wipper B Romero-Gutierrez C Winkelman J. Restless legs syndrome severity in the National RLS Opioid Registry during the COVID-19 pandemic. Sleep Med. 2022;90:96–101. 35131547 10.1016/j.sleep.2022.01.011 PMC8783980

[69] Morais M Franco B Holanda A. PTPRD as a candidate druggable target for therapies for restless legs syndrome? J Sleep Res. 2021;30:e13216. 33111449 10.1111/jsr.13216

